# Differential Expression of Hypothalamic and Gill-*crh* System With Osmotic Stress in the Euryhaline Black Porgy, *Acanthopagrus schlegelii*

**DOI:** 10.3389/fphys.2021.768122

**Published:** 2021-11-11

**Authors:** Adimoolam Aruna, Tsan-Ping Wang, Jyun-Cing Cao, Dan-Suei Lan, Ganesan Nagarajan, Ching-Fong Chang

**Affiliations:** ^1^Department of Aquaculture, National Taiwan Ocean University, Keelung, Taiwan; ^2^Department of Basic Sciences, PYD, King Faisal University, Al Hofuf, Saudi Arabia; ^3^Center of Excellence for the Oceans, National Taiwan Ocean University, Keelung, Taiwan

**Keywords:** *crh*, *crhr*, α*-nka*, gill, fish, brain, osmotic stress

## Abstract

The local gill production of corticotropin releasing hormone (*crh*) and *crh*-receptor (*crhr*) is hypothesized to play important roles during seawater (SW) and freshwater (FW) acclimation in euryhaline black porgy (*Acanthopagrus schlegelii*). The mRNA expression of *crh*, *crhr*, and *Na*^+^/*K*^+^*-ATPase* (*a-nka*) was examined in SW and FW diencephalon (Dien) and in the gills at different exposure time by Q-PCR analysis. The *in situ* hybridization results indicate that *crh* mRNA hybridization signals were more abundant in FW fish in the gigantocellular (PMgc) and parvocellular (PMpc) part of the magnocellular preoptic nucleus versus SW fish. The *crh* and *crhr-*expressing cells were located in basal cells of gill filament. Furthermore, *in vitro* dexamethasone (DEX) treatment could increase the *crh*-system in the gill. Increased transcripts of the *crh*-system in the gill via *in vitro* and *in vivo* CRH treatments suggest that CRH may regulate the system in a local manner. The a-Nka cells were localized in the filament and secondary lamellae mitochondria rich cells (MRCs) of FW fish at 8 h and 1 day. a-Nka cells were seen in both filament and lamellae in the FW but much less in SW fish indicating that gills play key roles in black porgy osmoregulation. Gill *crh* and *crhr* play important roles in the response to salinity stress.

## Introduction

Stress stimulates the corticotrophin releasing hormone (*crh*) and *crh*-receptor (*crhr*) from the hypothalamus and pituitary by activation of the hypothalamic pituitary interrenal (HPI/fish) and/or hypothalamic pituitary adrenal (HPA/mammals)-axis (Dautzenberg and Hauger, 2002). In vertebrates, *crh* is the dominant hypothalamic neuropeptide controlling the HPI-axis (Vale et al., 1981; Rivier and Vale, 1983). Stress acts in the nervous system and converges at the hypothalamus, and the final products are corticosteroid hormones, particularly, cortisol, which is believed to regulate homeostasis. Furthermore, this same corticosteroid terminates the stress response by interacting directly with the hypothalamus or anterior pituitary to attenuate *crh* and *crhr* production (Slominski et al., 2000).

In mammals, the skin is a barrier between the external environment and the internal milieu and plays a critical role in maintaining internal homeostasis. Slominski and Wortsman (2000) suggest that the skin has a high sensory capability for stress stimuli and is tightly coupled to a local response system. During stress, the mammalian skin expressed *crh* and *crhr* and is highly reactive to common stressors such as immune cytokines, ultraviolet radiation, cutaneous pathology, or even the physiological changes associated with the hair cycle phase (Slominski et al., 2001). However, the body surfaces of teleosts are continuously exposed to an aquatic environment and are in intimately physiological contact with fish via the body fluid compartments and the epithelium of the gill, kidney, and intestine (Flik et al., 2006). Physiological processes, such as gas exchange, osmoregulation, excretion of nitrogenous waste products and acid-base balance, take place in the osmoregulatory organs (Evans et al., 2005). Thus, damage to these organs could result in the compromise of fish survival.

The gill is the only organ that is diffused by the entire cardiac output and has an extensive vascular surface area in contact with the plasma (Olson, 1998). The gills also play a dominant role in endocrine regulation. They are an endocrine target and are metabolically active tissue (Evans et al., 2005). The teleost gill possesses two morphologically distinct epithelia. One is a multilayered filament epithelium with pavement, mucous, and mitochondria rich cells (MRCs) which is largely involved in ion exchange. Another is a bi-layered lamellar epithelium made of undifferentiated, myoepithelial-like, granular, and neuroendocrine cells involved in gas exchange between the blood and the ambient water (Li et al., 1995). Versus terrestrial vertebrates, aquatic fish face a more challenging task to achieve an internal homeostasis due to the presence of ionic and/or osmotic gradients that are hostile to the body fluids of fish (Kaneko et al., 2002; Kaneko and Hiroi, 2008).

Black porgy, *Acanthopagrus schlegelii*, is an euryhaline teleost that maintains ion and water balances in a wide variety of environmental salinity via osmoregulatory mechanisms (Tomy et al., 2009). Thus, in fish, the gill is an important osmoregulatory organ that creates ionic and osmotic gradients between the body fluid and external environments (Marshall and Grosell, 2006). In the gill, MRCs or chloride cells present in gill epithelia are a major site of ion secretion and absorption. They are important in SW and FW adaptation, respectively (Kaneko et al., 2008). The chloride cells or MRCs are structurally characterized by many mitochondria including an abundant tubular system where ion-transporting enzymes such as Na^+^/K^+^-ATPase, Ca^2+^-ATPase, and Ca^2+^/Na^+^ exchanger is located (Wendelaar Bonga et al., 1990; Flik and Verbost, 1993; Marshall, 2002). The MRCs are the site of the active Ca^2+^ transport that underlies transepithelial Ca^2+^ uptake in both freshwater (FW) and seawater (SW) fish (Marshall et al., 1992; McCormick et al., 1992; Verbost et al., 1994).

Here, we hypothesize that the gill is an essential vital organ that directly faces the exterior and has an endocrine-like function to acclimate and adapt to environmental changes. To maintain homeostasis, a gill *crh* system (*crh*/*crhr*) may be activated by external stressors including *in vivo* and/or *in vitro* conditions. Locally produced *crh* and *crhr* in the black porgy gill can respond to ambient salinity stress versus the hypothalamic *crh-*system. Based on limited information available on the expression of *crh* and *crhr* in the gill of common carp, *Cyprinus carpio*, we study here the expression of *crh*, *crhr*, and Na^+^/K^+^-ATPase (*a-nka*) in the black porgy diencephalon (Dien) and gill by Q-PCR analysis (Mazon et al., 2006). This is also the first study to localize the transcripts of *crh* and *crhr* in SW and FW-acclimated black porgy gill at day 1 and day 30 by *in situ* hybridization. We further performed *in vitro* and *in vivo* dexamethasone (DEX) and CRH treatment to analyze the mRNA expression pattern of *crh*-system in Dien and gill. Immunohistochemical studies with a-Nka antibody have identified the a-Nka immunoreactivity (ir) cells in the gill at day 1 and day 30 during SW and FW acclimation.

## Materials and Methods

### Experimental Fish

Black porgy (6–7 months old, *n* = 190) (body weight = 17.69 ± 0.59 g, body length = 9.96 ± 0.37 cm) were procured from an aquaculture farm of southern Taiwan (Chiayi County) and allowed to acclimated for 1 month at the National Taiwan Ocean University aquarium in seawater (33 ppt) with a natural light system. The water temperature, pH and dissolved oxygen (DO) values of which ranged from 19 to 24°C, 8.11 to 8.18, and 7.7 to 7.8 mg/L, respectively. Seawater was obtained from the open sea and filtered through sand. Freshwater was obtained from the tap and was put in the tank for 1 week with aeration for dechlorination (pH and DO values were 7.91–8.08 and 7.8–8.0 mg/L, respectively). The fish tanks were aerated to maintain adequate dissolved oxygen. The water was continuously aerated through air stones. The level of the aeration was sufficient to sustain dissolved oxygen levels for all experiments. The sponge filters and gravel were used to filter the solids from fish waste and uneaten feeds. Fish were fed pelleted dry food *ad libitum* at a daily ration of 1% of their estimated body weight. For each experiment, the fish were anesthetized with 1% glycophenol monophenyl ether and decapitated. The diencephalon and gill samples were collected and snap frozen in liquid nitrogen at −80°C. All experiments were conducted in accordance with the principles and procedures approved by the Institutional Animal Care and Use Committee, National Taiwan Ocean University, Taiwan (# 99026).

### Experimental Design-Experiment 1

#### Seawater to Freshwater Transfers at Different Time Exposure

To characterize the endocrine changes in osmoregulation in response to acute salinity stress, an experiment was conducted where fish were randomly divided into two groups and maintained in SW (33 ppt). After an initial acclimation period (30 days), fish were maintained in SW (*n* = 40) and transferred to FW (0 ppt). Respective control groups (*n* = 40) received the SW with no salinity changes. The diencephalon and gill samples (*n* = 10) were collected after 8 h, 1 day, 4 days, and 30 days of transfer.

### Experimental Design-Experiment 2

#### *In vivo* Treatment of *crh*

The fish (*n* = 24) were cultured in 2,000 L tanks for 2 weeks. After an initial acclimation, the fish were randomly divided into three groups (*n* = 8 per group). The different doses of mammalian *crh* (Sigma, St. Louis, MO, United States) (10 and 40 μg/kg) were prepared by dissolving *crh* with phosphate buffered saline (PBS) (pH 7.4). Fish were injected intraperitoneally with *crh* twice on day 1 and day 4. Control fish were injected with coconut oil and treated as a control. We collected the samples on day 5.

### Experimental Design-Experiment 3

#### *In vitro* Gill Culture With *crh* and Dexamethasone

The fish (*n* = 64) were cultured in 2,000 L tanks for 2 weeks. After an initial acclimation, the fish were randomly divided into eight groups (*n* = 8 per group). The serial dilutions of mammalian DEX (low 10^–8^ M, medium 10^–6^ M, and high 10^–4^ M) and *crh* (low 10^–9^, medium 10^–7^, and high 10^–5^ M) were prepared and the gill filaments were incubated for 2 and 4 days with the respective concentrations. A fish group was incubated with L-15 medium as a control. After *in vitro* culture, gill filaments were removed with forceps and then snap frozen in liquid nitrogen at −80°C until RNA isolation.

### Primary Gill Culture

Primary gill filaments were excised from the arches and separated from one another. Five to six primary gill filaments were placed in each petri dish (*n* = 8 fish, each fish per petri dish) containing 1% penicillin-streptomycin in Leibovitz L-15 medium (Gibco, United States), this was incubated in ice for an hour. After incubation, the filaments were washed with 1X PBS to remove blood. The gill filaments were again rinsed with 5% fungizone in 1X PBS. Again, the filaments were rinsed with 5% penicillin-streptomycin in L-15 medium. Serial dilutions of DEX (10^–8^, 10^–6^, and 10^–4^ M) and CRH (10^–9^, 10^–7^, and 10^–5^ M) were prepared in L-15 medium. The incubation medium was removed and replaced with the serial dilutions of CRH, DEX, and/or vehicle in L-15 medium containing 50 units ml^–1^ penicillin and 50 μg ml^–1^ streptomycin equilibrated with a 99% oxygen and 1% carbon dioxide gas mixture. Gill filaments were incubated at 15°C for 48 h (day 2) and 96 h (day 4) in a humidified chamber with 99%:1% of O_2_:CO_2_ with gentle shaking. After culture, gill filaments were removed with forceps and then snap frozen in liquid nitrogen at −80°C until RNA isolation.

### RNA Extraction, First-Strand cDNA Synthesis and Cloning

RNA was isolated from the Dien and gill by using TRIzol^®^ reagent and reverse transcribed (Gibco BRL, Grand Island, NY, United States) according to the protocol of the manufacturer. The resulting cDNA was used as a template for the subsequent PCR amplification of the genes used here.

Genes involved in the stress response were cloned from cDNA of the black porgy gill. Multiple alignments of previously published sequence of the respective genes were constructed using CLUSTAL X program (version 1.81) to find the conserved region. Primers were designed from here (Table 1). PCR reactions were performed with 2.5 μl of 10X reaction buffer [200 mM Tris–HCl (pH 8.4), 500 mM KCl], 1 μl of 10 mM dNTP, 1 μl of 2 mM MgCl_2_, 0.5 μl each of 10 μM sense and antisense primers, 1 μl cDNA, and 0.2 μl superscript enzyme (Invitrogen, Carlsbad, CA, United States) in a final volume of 25 μl. The PCR conditions were set as follows: 94°C for 5 min, 94°C for 30 s, 50°C for 30 s, 72°C for 30 s for 35 cycles, and 72°C for 10 min. The PCR products were verified by electrophoresis on a 1.5% agarose gel and visualized using ethidium bromide staining DNA fragments were excised using a Gel-M^TM^ Gel Extraction system Kit (Bio 101) (Viogene, La Jolla, CA, United States) and cloned into pGEM^®^ – T Easy vector (Promega, Madison, WI, United States). Plasmids containing the insert were sequenced using a dye terminator cycle sequencing kit (Perkin Elmer, Foster City, CA, United States) and submitted to the Basic Local Alignment Search Tool (BLAST) for making comparison with the known sequences accessible in NCBI database.

**TABLE 1:** List of primers used for RT-PCR, Q-PCR, and *in situ* hybridization analysis.

**Gene**	**Orientation**	**Nucleotide sequence (5′ – 3′)**	**Usage**	**Accession no.**
*Crh*	F	5′-GGCGGATCACCTGCGATCT-3′	RT-PCR	FJ445422
	R	5′-GATCTGACCTTCCACCTGC-3′	RT-PCR	
	F	5′-CAGCTCCCCAAACCCAAAA-3′	Q-PCR	
	R	5′-CCAAGCCGCTCCAGGAT-3′	Q-PCR	
	S	5′-CCGCTACGAATGTCGGGCTATTGAG-3′	*in situ*	
	AS	5′-CTTCCCCTCTCCATCGAGTC-3′	*in situ*	
*crh-r*	F	5′-CCACCACGTCAGAGACCAT-3′	RT-PCR	FJ445423
	R	5′- GTTTTTGGAGTCCTTCCAGGG-3′	RT-PCR	
	F	5′- AAGAAGTTGGTGGAGTGGAAATAGTT-3′	Q-PCR	
	R	5′-GAGGTGCACGAAAGCAACGT-3′	Q-PCR	
	S	5′-GGAACCTCATCACCGCCTTCATC-3′	*in situ*	
	AS	5′- CCCCAGGAGAGGGAGAAGAAC-3′	*in situ*	
*a-nka*	F	5′-ACCGTGGCCCACATGTG-3′	RT-PCR	EF621407
	R	5′-GGTCCCGCTCTGGTTCTCA-3′	RT-PCR	
	F	5′-ACCGTGGCCCACATGTG -3′	Q-PCR	
	R	5′-GGTCCCGCTCTGGTTCTCA -3′	Q-PCR	
*gapdh*	F	5′-AGGCTTCCTTAATCTCAGCATAAGAT-3′	RT-PCR	DQ399798
	R	5′-GGTGCCTGTGGCTGATGTG -3′	RT-PCR	
	F	5′-GCATCTTGCACGGCTAACT -3′	Q-PCR	
	R	5′-CGGCGCCGGCATCGAAGAT -3′	Q-PCR	

*F, Forward primer; R, Reverse primer; *in situ*, *in situ* hybridization.*

### Tissue Distribution mRNA Expression of *crh*, *crhr*, and *α-nka* in Dien and Gill

Total RNA was extracted from the Dien and gill of black porgy (*n* = 3). The complementary DNA (cDNA) strand was synthesized with 1 μg of total RNA using SuperScript II with the oligo(dT)_12__–__18_ primers. Gene specific primers were employed for RT-PCR analysis (Table 1). The *glyceraldehyde 3-phosphate dehydrogenase* (*gapdh*) was used as an internal control. The *gapdh* transcripts did not significantly change between or among the treatments. The PCR conditions were as follows: 94°C for 1 min, 55°C for 1 min, and 72°C for 2 min for 30 cycles and the PCR products were separated on 1.5% agarose gel. Reactions in which reverse transcriptase was neglected were used as negative controls for SW and FW fish.

### Q-PCR Analysis

Q-PCR analysis was performed to analyze the gene expression of *crh*, *crhr1*, and α*-nka* in the Dien and gill during FW and SW acclimation with iQ^TM^ Multicolor Real Time-PCR Detection system (Bio-Rad). Primers were designed using primer expression software (Applied Biosystems, Foster City, CA, United States) (Table 1). Gene quantification of standards, samples, and controls was conducted simultaneously in a Q-PCR machine (iQ^TM^ Multicolor Real-Time PCR Detection System; Bio-Rad Co., Hercules, CA, United States) with iQ^TM^ SYBR green (Bio-Rad) as a dsDNA minor-groove binding agent, forward and reverse primers, and water according to our previous study (Aruna et al., 2012a). Calculation of PCR efficiency was based on the slope of the relationship between log input cDNA (transcript concentrations) vs. cycle threshold (Ct). We used an efficiency-corrected method to calculate the relative expression level of *crh*, *crhr*, and a*-nka* in the hypothalamus and the gill. The correlation of the standard curve was −0.999. The *gapdh* was used as a control gene to calibrate the mRNA expression level, because there were no significant changes in the expression levels between all controls and experimental groups.

### *In situ* Hybridization

The *in situ* hybridization was carried out to localize the *crh* and *crhr* transcripts in the hypothalamus and gill of black porgy. The tissues were fixed in a 4% paraformaldehyde in phosphate-buffered saline (PBS) at 4°C overnight and embedded in paraffin. Paraffin sections (5 μm) were collected on TESPA-treated slides (3-aminopropyltriethoxysilane, Sigma). For *in situ* hybridization, the digoxigenin (DIG)-labeled sense and anti-sense RNA probes of *crh* and *crhr* (primers in Table 1) were prepared in black porgy using T7 and Sp6 polymerase (Promega, Madison, WI, United States) on linear plasmid DNA containing respective inserts of the genes in the pGEM-T Easy vector in accordance with the methods described previously (Aruna et al., 2012a). The resulting PCR products purified (Viogene, Bio 101, La Jolla, CA, United States) and the quantity of the PCR amplification product was measured via spectrophotometry at 260 nm. The RNA probe quality was checked by spectrophotometry at 260 nm. The preparation of RNA probe by PCR amplification was described previously (Thisse and Thisse, 2008).

The serial sections proceeded to rehydration, prehybridization, and hybridization according to our previous study (Aruna et al., 2012a). The sections were incubated with an alkaline phosphatase-conjugated sheep anti-digoxigenin antibody (Roche, Penzberg, Germany) (dilution 1:2,000 in 2% blocking reagent) overnight at 4°C. The hybridization signals were visualized by NTMT (100 mM NaCl, 100 mM Tris–HCl, pH 9.5, 50 mM MgCl_2_, 0.1% Tween-20) and NBT/BCIP staining which was stopped by washing the sections with water.

### Immunohistochemistry

Immunohistochemical analysis of the a-Nka was performed in the gill of black porgy. A few deparaffinized serial sections (5 μm) were incubated with 3% H_2_O_2_ in PBS. The sections were then incubated with 1.5% normal goat serum for 30 min and with commercial primary antibody (a-Nka-IgG) (Santa Cruz Biotechnology Inc., Santa Cruz, CA, United States) overnight at 4°C. This was followed by incubation with anti-rabbit IgG (Vector Laboratories Inc., Burlingame, CA, United States) for 1 h at room temperature. The sections were then visualized by an ABC kit (avidin-biotin, Vector Laboratories Inc.) and DAB (3,3′-diaminobenzidine, Sigma).

### Statistical Analysis

Data are expressed as means ± standard error of the mean. The values were analyzed by one-way ANOVA, followed by Student–Newman–Keuls multiple tests. Value with *p* < 0.05 indicating a significant difference and are denoted by “a” and “b.” Student’s *t*-test was also conducted to determine significant differences (*p* < 0.05) between SW and FW fish denoted by asterisks (^∗^).

## Results

### Tissue Distribution of *crh*, *crhr*, and *a-nka*

**FIGURE 1:**
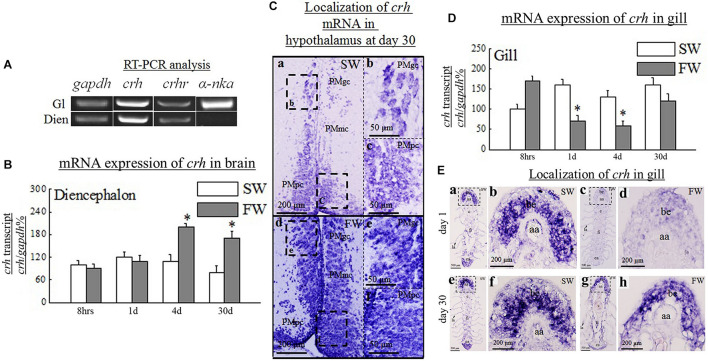
**(A)** The black porgy corticotropin releasing hormone (*crh*), crh receptor (*crhr*), and a-*nka* cDNA consisted of 363 bp, 556 bp, and 968 bp. A single band of the expected size was obtained for *crh, crhr*, and α*-nka* mRNA in the tissue analyzed by RT-PCR. *crh* and *crhr* mRNA were detected in the diencephalon (Dien) and gill. *a*-*nka* mRNA was strongly detected in the gill. In Dien, the intensity of the band was very weak. The *gapdh* was used as an internal control. **(B)** Comparative analysis of *crh*, gene expressions as determined by quantitative real-time PCR in Dien of black porgy from a seawater (SW) to freshwater (FW) transfer at 8 h, day 1, day 4, and day 30. Each data had 10 fish. **(C)** Localization of *crh* in the hypothalamus gigantocellular (PMgc) **(b,e)** and parvocellular part of the magnocellular preoptic nucleus (PMpc) **(c,f)** was performed by *in situ* hybridization during SW **(a–c)** and FW **(d–f)** acclimation of black porgy on day 30. The representative fish is shown from one of 3 fish. There was no notable signal found in the representative sense pictures (not shown). PMgc, gigantocellular part of the magnocellular preoptic nucleus; PMmc, magnocellular part of the magnocellular preoptic nucleus; PMpc, parvocellular part of the magnocellular preoptic nucleus. **(D)** Comparative analysis of *crh*, gene expressions as determined by quantitative real-time PCR in gill of black porgy from SW to FW at 8 h, day 1, day 4, and day 30. Data (10 fish for each data) are expressed as means ± standard error of the mean. Student’s *t* test was conducted to determine significant differences (*p* < 0.05) between SW and FW fish are denoted by asterisks (^∗^). **(E)** The *in situ* hybridization was performed to localize *crh* transcripts in black porgy gill during SW and FW acclimation at day 1 **(c,d)** and day 30 **(g,h)**. The transcripts of *crh* hybridization signal were weak in FW acclimated fish **(c,d)** in the branchial epithelium when compared to the SW acclimated fish **(a,b)** at day 1. And, the hybridization signals of *crh* were similar between SW **(e,f)** and FW **(g,h)** fish at day 30 in the branchial epithelium of the gill. The representative fish is shown from one of 3 fish. There was no notable signal found in the representative sense pictures (not shown). aa, afferent artery; be, branchial epithelium; ea, efferent artery; fi, filament; la, lamellae.

The expected single band of *crh*, *crhr*, and *a-nka* was obtained in all the examined tissues except *a-nka* in Dien by RT-PCR, though with varying intensity in expression. A strong band of *crh* and *crhr* was found in the Dien and gill. In the gill, the expression of *a-nka* was strong, but no expression was observed in Dien (Figure 1A). *crh*, *crhr*, and a-*nka* mRNA levels were determined by RT-PCR in Dien and gill of SW and FW black porgy. *gapdh* was used as an internal control (Figure 1A).

### Q-PCR Analysis of *crh* and *crhr* mRNA Expression in Dien During Freshwater Transfer

**FIGURE 2:**
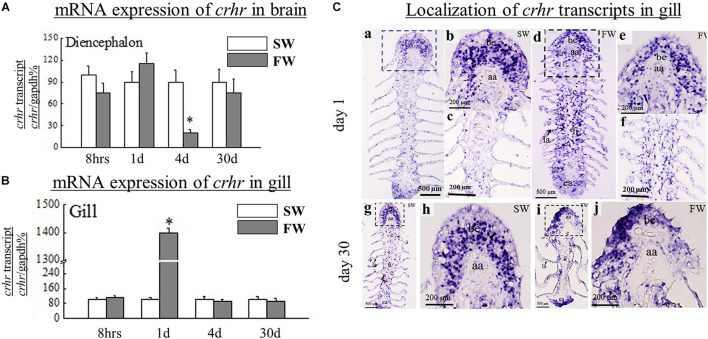
**(A)** Comparative analysis of *crhr*, gene expressions as determined by quantitative real-time PCR in diencephalon (Dien) of black porgy from SW to FW at 8 h, day 1, day 4, and day 30. Each data had 10 fish. **(B)** Comparative analysis of *crhr*, gene expressions as determined by quantitative real-time PCR in the gill of black porgy from a SW to FW transfer at 8 h, day 1, day 4, and day 30. Each data had 10 fish. **(C)** The *in situ* hybridization was performed to localize *crhr* transcripts in black porgy gill during SW and FW acclimation on day 1 **(a–f)** and day 30 **(g–j)**. The mRNA expression of *crhr* signals was more abundant throughout the gill in FW-acclimated fish **(d–f)** compared to the SW fish **(a–c)** on day 1. On day 30, the hybridization signals of *crhr* were almost similar between SW **(g,h)** and FW **(i,j)** fish in the branchial epithelium of the gill. The representative fish is shown from one of 3 fish. There was no notable signal found in the representative sense pictures (not shown). aa, afferent artery; be, branchial epithelium; ea, efferent artery; fi, filament; la, lamellae. Student’s *t*-test was conducted to determine significant differences (*p* < 0.05) between SW and FW denoted by asterisks (*).

The expression of *crh* in FW was highly expressed in Dien at day 4 and day 30 versus the control (SW) fish (Figure 1B). In contrast, the expression of *crhr* (Figure 2A) was significantly (*p* < 0.05) downregulated in the Dien at day 4 FW when compared to their respective controls. No significant difference between SW and FW was observed in the expression of *crhr* in Dien at 8 h, day 4, and day 30 (Figure 2A).

### Q-PCR Analysis of *crh*, *crhr*, and *a-nka* mRNA Expression in Gill During Freshwater Transfer

**FIGURE 3:**
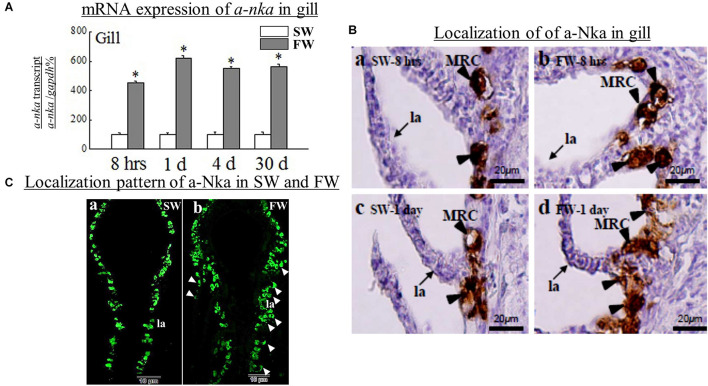
**(A)** Comparative analysis of *a-nka*, gene expressions as determined by quantitative real-time PCR in diencephalon (Dien) of black porgy from SW to FW at 8 h, day 1, day 4, and day 30. Each data had 10 fish. **(B)** The immunohistochemistry was performed to localize the a-Nka cells in black porgy gill during SW and FW acclimation on day 1 and day 30. The expression of a-Nka in the gill was increased in FW at 8 hrs (b) and day 1 (d) compared to the respective SW fish **(a,c)**. The Nka was expressed in the filaments and lamellae in the FW fish **(b,d)** but the a-Nka was expressed only in the gill filament not lamellae in SW fish **(a,c)**. The representative fish is shown from one of 3 fish. There were no notable signals found in the control (not shown). **(C)** Localization pattern of a-Nka cells during SW **(a)** and FW **(b)** of black porgy. The a-Nka cells were also appeared in the lamellae (pointed with arrow heads) in addition to filament in FW fish **(b)**. MRC: mitochondria rich cell, fi: filament, la: lamellae. Student’s *t*-test was conducted to determine significant differences (*p* < 0.05) between SW and FW denoted by asterisks (*).

A significant (*p* < 0.05) reduction was found in the gill *crh* at day 1 and day 4 in FW versus SW (Figure 1D). In contrast, the *crhr* mRNA levels were significantly (*p* < 0.05) increased in gill at day 1 in FW versus SW (Figure 2B). The expression levels of α*-nka* were 5, 6, 4.5, and 4.4-fold higher in FW at 8 h, day 1, day 4, and day 30, respectively, versus controls (Figure 3A). No significant difference was observed in the expression of *crh* and *crhr* in gill between SW and FW fish at 8 h and day 30 (Figures 1D, 2B).

### Localization of *crh* Transcripts in Hypothalamus at Day 30 by *in situ* Hybridization

The localization of *crh* transcripts was performed in the hypothalamus at day 30 SW (Figures 1Ca–c) and FW (Figures 1Cd–f) by *in situ* hybridization. The *crh* transcript hybridization signals were detected in both SW (Figure 1Ca) and FW fish (Figure 1Cd). The *crh* mRNA hybridization signals were more abundant in FW fish in the gigantocellular part of the magnocellular preoptic nucleus (PMgc) (Figure 1Ce) and parvocellular part of the magnocellular preoptic nucleus (PMpc) (Figure 1Cf) versus the SW fish (Figures 1Cb,c).

### Localization of *crh* and *crhr* Transcripts in Gill at Day 1 and Day 30 by *in situ* Hybridization

The expression of *crh* (Figure 1E) and *crhr* (Figure 2C) gene was examined by *in situ* hybridization in gill at day 1 and day 30. The hybridization signals of *crh* and *crhr* mRNA in the gill were located near and around the afferent filamental artery (Figures 1Ea–h, 2Ca–j). The transcripts of *crh* hybridization signals were decreased in FW acclimated fish in the branchial epithelium (Figures 1Ec,d) versus the SW acclimated fish (Figures 1Ea,b) at day 1. In contrast, the mRNA expression of *crhr* signals was more abundant throughout the gill in the FW acclimated fish (Figures 2Cd–f) versus the SW fish (Figures 2Ca–c) at day 1. The hybridization signals of *crh* and *crhr* were similar between SW (Figures 1Ee,f, 2Cg,h) and FW (Figures 1Eg,h, 2Ci,j) fish at day 30 in the branchial epithelium of the gill.

### Immunohistochemistry Analysis of a-Nka Antibody in Gill Tissue

The expression of a-Nka was examined in the gill (Figures 3Ba–d). The a-Nka cells were mainly expressed in the MRCs. The expression of a-Nka genes in the gill was increased in FW at 8 h, day 1, day 4, and day 30. However, we performed the immunohistochemistry at 8 h and day 1 in both SW (Figures 3Ba,b) and FW (Figures 3Bc,d). The a-Nka cells were expressed in the filaments and lamellae in the FW fish at 8 h and day 1 (Figures 3Bb,d) but the a-Nka cells were expressed only in the gill filament but not lamellae in SW fish (Figures 3Ba,c). The fluorescent hybridization showed the pattern of a-Nka cells in SW and FW fish (Figures 3Ca,b). The a-Nka cells were localized in the filament at SW (Figure 3Ca). However, FW fish had a-Nka cells localized in both filament and lamellae (Figure 3Cb).

### Non-colocalization of *crh*/*crhr* Transcript Cells With *a-nka* Cell in Gill Filament

**FIGURE 4:**
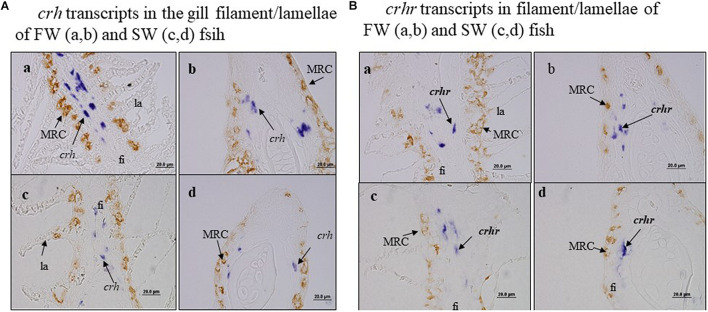
There was no co-localization of *crh* transcript cells vs. a-Nka cells and *crhr* transcript cells vs. *a-nka* cells in the gill filament of FW and SW fish. Fi, filament; la, lamellae. **(A)** *crh* transcripts in the gill filament/lamellae of FW **(a,b)** and SW **(c,d)** fish. **(B)** *crhr* transcripts in filament/lamellae of FW **(a,b)** and SW **(c,d)** fish.

We further examined the co-localization of *crh* transcript cells vs. a-Nka cells and *crhr* transcript cells vs. a-Nka cells in the gill filament or lamellae of FW and SW fish by ISH and IHC (Figure 4). Our data found that *crh* and *crhr* transcript cells were not co-localized with a-Nka cells in either FW or SW fish (Figure 4). The *crh* and *crhr* cells are suggested to localize in the basal cells of the filament and a-Nka cells are localized in the filament epithelium (Figure 4).

### Q-PCR Analysis of *crh*, *crhr*, and *a-nka* mRNA Expression in Dien and Gill During *in vivo* CRH Treatment

**FIGURE 5:**
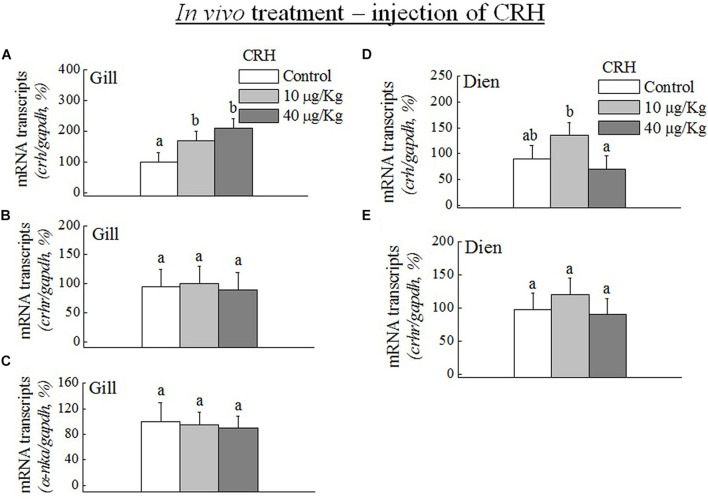
Q-PCR analysis was performed to examine the mRNA expression of *crh*, *crhr*, and *a-nka* in the gill **(A–C)** and diencephalon (Dien) **(D,E)** during *in vivo* CRH treatment with low (10 μg/kg) and high (40 μg/kg) doses. Data (*n* = 8 for each data) are expressed as means ± standard error of the mean. The values were analyzed by one-way ANOVA, followed by a Student–Newman–Keuls multiple test, with *p* < 0.05 indicating a significant difference, are denoted by “a” and “b.”

The transcripts of *crh* were significantly (*p* < 0.05) increased in the gill *in vivo* at low (10 μg/kg) and high doses (40 μg/kg) of *crh* (Figure 5A). No significant (*p* > 0.05) difference was observed in the mRNA expression of *crhr* (Figure 5B) and a*-nka* (Figure 5C) in the gill *in vitro* at both low and high doses of CRH. No significant (*p* > 0.05) difference was found in the transcripts of *crh* and *crhr* in the Dien at low and high doses of *in vivo crh* treatment (Figures 5D,E).

### Q-PCR Analysis of *crh* and *crhr* mRNA Expression in Gill During *in vitro* CRH Treatment

**FIGURE 6:**
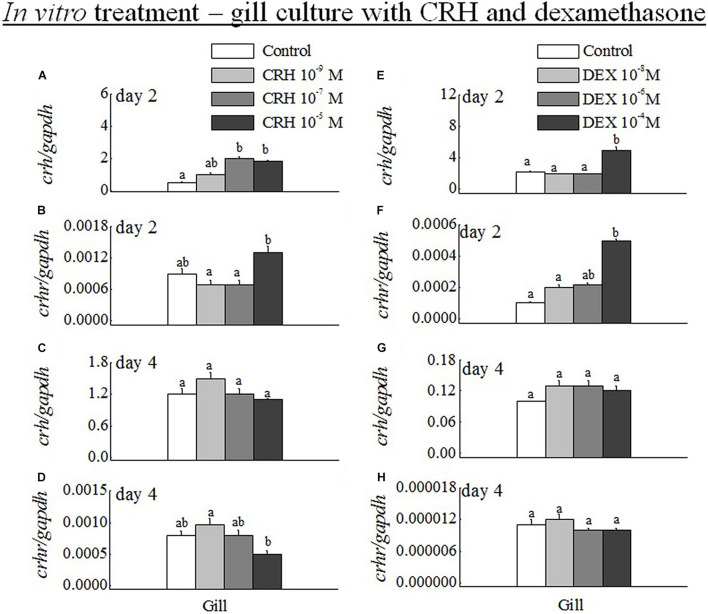
**(A–D)** Q-PCR analysis was performed to examine the mRNA expression of *crh* and *crhr* in gill on day 2 **(A,B)** and day 4 **(C,D)** during *in vitro* gill culture with CRH low (10^–9^ M), medium (10^–7^ M) and high (10^–6^ M) doses. Data (*n* = 8 for each data) are expressed as means ± standard error of the mean. The values were analyzed by one-way ANOVA, followed by a Student–Newman–Keuls multiple test, with *p* < 0.05 indicating a significant difference, and are denoted by “a” and “b.” **(E–H)** Q-PCR analysis was examined the mRNA expression of *crh* and *crhr* in gill on day 2 **(E,F)** and day 4 **(G,H)** during *in vitro* gill culture with DEX low (10^–8^ M), medium (10^–6^ M), and high (10^–4^ M) doses. Data (*n* = 8 for each data) are expressed as means ± standard error of the mean. The values were subjected to analysis by one-way ANOVA, followed by a Student–Newman–Keuls multiple test, with *p* < 0.05 indicating a significant difference, are denoted by “a” and “b.”

The transcripts of *crh* were significantly increased at medium (10^–7^ M) and high (10^–5^ M) doses of CRH (Figure 6A). The *crhr* was significantly (*p* < 0.05) increased only at high dose in the gill on day 2 (Figure 6B). The long duration of gill culture with CRH did not change the mRNA levels of CRH on day 4 (Figure 6C). However, high dose of *crh* significantly (*p* < 0.05) downregulated the *crhr* mRNA expression in the gill on day 4 (Figure 6D). In addition, the low dose (10^–9^ M) of CRH did not change the *crh* and *crhr* transcripts in the gill on day 2 and day 4 (Figures 6A–D).

### Q-PCR Analysis of *crh* and *crhr* mRNA Expression in Gill During *in vitro* Dexamethasone Treatment

The mRNA expression of *crh* (Figure 6E) and *crhr* (Figure 6F) was significantly (*p* < 0.05) increased at high dose (10^–4^ M). No significant (*p* > 0.05) difference was observed in the gill at low (10^–8^ M) and medium (10^–6^ M) doses of DEX versus their controls at day 2. In addition, there was no significant (*p* > 0.05) difference in the transcripts of *crh* (Figure 6G) and *crhr* (Figure 6H) at low, medium, and high dose of DEX at day 4.

## Discussion

We show that the mRNA expression of *crh*, *crhr*, and *a-nka* in the Dien and gill of black porgy during a SW to FW acclimation at 8 h, day 1, day 4, and day 30. In addition, we localized *crh* and *crhr* transcripts in SW and FW Dien and gill of black porgy by *in situ* hybridization at day 1 and day 30. The *crh* (*in vitro*, *in vivo*) and DEX (*in vitro*) treatments with different doses were performed to analyze the differential mRNA expression pattern of *crh* and *crhr* in Dien and gill. Furthermore, we localized the a-Nka cells in FW and SW-acclimated black porgy gill at day 1 and day 30 by immunohistochemistry. The mRNA expression of *crh*, *crhr*, and *a-nka* transcripts during FW transfer and *in vivo* and *in vitro crh* and DEX treatment at different days with different doses were elaborately framed in the current study.

The serum osmolality levels were significantly decreased in the FW black porgy at day 1 and 30 (unpublished data). In the SW fish, serum osmolality was significantly higher compared to the FW fish, as indicated in our previous study (Tomy et al., 2009). Serum cortisol was significantly increased at day 1 and returned to normal levels at 30 (unpublished data). According to this, it seems that black porgy may require only a short period for acclimation to osmotic stress. The total concentration of solutes such as the inorganic ions present in the fluid is the osmolality (Soegianto et al., 2017). However, at the time of initial acclimation the serum osmolality levels were significantly different between SW and FW fish; this reflects the acclimation. The euryhaline teleost reduced their ion secretion rapidly when quickly moved to a FW environment. Overall, the black porgy maintained its serum osmolality within the range of 270–420 mOsmol kg^–1^. This suggests efficient hyper or hypo osmoregulation in black porgy (Tomy et al., 2009).

Generally, the teleost *crh* system was activated through the HPI-axis during the initial stress (Huising et al., 2004; Doyon et al., 2005). The expression of *crh* could regulate itself in response to the stress induced by the elevation of glucocorticoids and neurogenic signals (Lachize et al., 2009). The increased cortisol levels could affect gill function via regulating gill ion transporters (Kiilerich et al., 2007). The elevated hypothalamic *crh* markedly downregulated the pituitary *crhr* expression because continuous release of the hypothalamic *crh* desensitized the pituitary corticotrope cells (Huising et al., 2004). Furthermore, repeated or chronic stress can increase the *crh* mRNA levels in mammalian hippocampus, paraventricular nucleus, and tilapia forebrain and hypothalamus (Makino et al., 1995; Aruna et al., 2015). This suggests that the intensity and duration of the salinity stress are important external factors regulating the brain *crh* system (Doyon et al., 2005; Aruna et al., 2012a).

Seawater and FW acclimation activated *crh*, *crhr*, and *a-nka* transcripts in the black porgy hypothalamus and gill. In agreement with Doyon et al. (2005), we show here that the expression of *crh* mRNA was significantly increased at day 4 and day 30 whereas the *crhr* transcripts were significantly downregulated at day 4 in the Dien of the black porgy. These data suggest that the transiently elevated levels of plasma cortisol may exert negative-feedback effects on the level hypothalamus (hypothalamic *crhr*) as suggested in carp pituitary (Huising et al., 2004). In addition, we detected the *crh* transcript hybridization signals in both SW and FW fish. Similar to Q-PCR data, the *crh* mRNA hybridization signals were more abundant in FW fish at PMgc and PMpc versus SW fish at day 30. The data suggest that the expression of *crh* mRNA is essential to the salinity stress.

Increased expression of *crhr* mRNA in the gill suggests that the gills are responsible for the stressors and regulate the ventilation rate and blood oxygen transport (Wendelaar Bonga, 1997; Mazon et al., 2006). Furthermore, the Q-PCR and *in situ* hybridization results show that the *crh* transcripts were significantly reduced in the black porgy gill at day 1 and day 4. Interestingly, the *crhr* was significantly increased in the gill at day 1. The local production of *crh* and/or *crhr* in the gill may be directly activated by external stressors.

There are very few studies on the expression and localization of *crh* and *crhr* in the gill during SW and FW acclimation. This is the first study in black porgy to localize the *crh* and *crhr* transcripts in SW and FW-acclimated gill at day 1 and day 30 by *in situ* hybridization. Similar to the Q-PCR results, the cellular transcripts of the *crh* and *crhr* were lower and higher in FW-acclimated fish, respectively. The transcripts of the *crh* and *crhr* were highly localized in the apical membrane of the branchial epithelium in both SW and FW black porgy. In addition, the *crhr* transcripts were localized in the basal cells of the gill filament as reported in common carp by immunohistochemistry (Mazon et al., 2006). Our previous studies showed glucocorticoid receptors and mineralocorticoid receptors in tilapia gill (Aruna et al., 2012b). Taken together, the expression and localization of *crh* and *crhr* in black porgy gill highlight that positive involvement of the *crh* system can balance the homeostasis during salinity stress.

The DEX (5 mg/kg)-treated pigs had no effect on the mRNA expression of *crh* in the hypothalamus after 3 h (Vellucci and Parrott, 2000). In mice, *in vitro* DEX treatment for 24–96 h caused a specific decrease in *crh* mRNA (Adler et al., 1988). Here, we elaborately framed out the mRNA expression of *crh* and *crhr* in the Dien and gill by *in vitro* and *in vivo crh* and DEX treatment. In contrast to Adler et al. (1988), a high *in vitro* dose of DEX increased the transcripts of the *crh* and *crhr* in black porgy gill on day 2. Pierson et al. (2004) showed that the high dose of DEX treatment leads to a chronic stress, which may activate the local *crh*-system in the gill.

We also found that the transcripts of *crh* were significantly increased in gill by *in vitro* and *in vivo* CRH treatment. *In vivo* CRH treatment did not cause any effects on the expression of *crh* and *crhr* transcripts in the black porgy Dien with different doses. Consistent with our findings, the *in vitro* and/or *in vivo* CRH treatment decreased and/or did not change the mRNA expression of *CRHR* in the mammalian anterior pituitary after acute or repeated immobilization stress (Hauger et al., 1977; Luo et al., 1995; Makino et al., 1995; Sakai et al., 1996; Roseboom et al., 2001). Sumitomo et al. (1987) and Yokoe et al. (1988) strongly suggested that the main source of plasma CRH was the hypothalamic paraventricular nucleus neurons with their terminals in the median eminence of mammals. In contrast to the mammals, the descending spinal *crh*-ir projections appear to be absent in teleosts (Lovejoy and Balment, 1999; Pepels et al., 2002). Taken together, the local gill *crh* system may respond more rapidly than the hypothalamic *crh* in black porgy.

The *crh* system is a powerful stress response tool in teleosts (HPI-axis/local *crh*). It can trigger cortisol release from the cascade signals of brain-pituitary to inter-renal gland to respond to the various stressors (Flik et al., 2006). Generally, the presence of *crh* in the brain (nucleus of preopticus) is associated with fear and anxiety (Majzoub, 2006). Pepels et al. (2002) found a *crh* in the heads of larvae of Mozambique tilapia 5 days post hatching, suggesting the importance of the *crh* system in teleosts. The cutaneous *crh* system may respond to external stressors in mammals (Slominski et al., 2001). We also found the changes in the expression of *crh-crhr* in the brain-pituitary and corticosteroid receptors in the gill during the stress response in tilapia (Aruna et al., 2012a,b, 2015). Gill tissue separates the internal and external environment and is constantly activated by external stressors or internal factors. The presence of local *crh*, *crhr*, and corticosteroid receptors in the gill may be important to respond to stress in fish. Thus, the activation of the local gill *crh-crhr* system may be regulated by the circulation (*crh*-*acth* system from the brain-pituitary, and cortisol from interrenal gland) or local *crh* during stress.

Consistent with the physiological model for ion secretion by gill epithelia, the FW transfer significantly increased the mRNA expression of *a-nka* at 8 h, day 1, day 4, and day 30 in black porgy gill. These findings suggest that gills actively adapt to FW with possible functional plasticity along with more rapid responses to environmental changes. Similar elevation in branchial a-*nka* mRNA transcripts has been reported in *Anguilla anguilla* (Cutler et al., 1995) and *Salmo trutta* (Madsen et al., 1995) for the transfer from FW to SW. Black porgy was used for the transfer from SW to FW (Tomy et al., 2009). During the SW to FW transfer, the expression of a-Nka was on the filaments and lamellae MRCs on day 1 in FW fish. On day 30, the a-Nka cells were increased in number and larger but were expressed only in the filament of FW fish when compared to the respective SW fish. These data suggest that the changes in the ionic composition of hypotonic media can induce dramatic modifications in the functions of the gills and alter the morphology of MRCs (Laurent et al., 1985; Perry and Laurent, 1989; Perry et al., 1992; Lee et al., 1996). Thus, there may be two types of MRC. A type whose function rules out ions in a marine environment and another type that absorbs Na^+^, Cl^–^, Ca^2+^ in a FW environment (Perry, 1997; Goss et al., 2001; Marshall, 2002). These MRCs were located along the filamental epithelium for many species and along the secondary lamellae (Wendelaar Bonga and van der Meij, 1989; Katoh and Kaneko, 2003; Carmona et al., 2004; Lima and Kültz, 2004; Allen and Cech, 2007). Consistent with these results, MRCs have been shown to localize on both the lamellae and filaments in hypo-osmotic environment (Cataldi et al., 1995; McKenzie et al., 1999). Furthermore, the expression and localization of *crh*, *crhr* and a-Nka cells in the gill showed that the brain-gill *crh* system might be involved in the osmoregulation.

## Conclusion

This study demonstrated *crh* and *crhr* in the gill by *in situ* hybridization during SW and FW in black porgy. The differential expression pattern of *crh* mRNA was found in Dien and gill during FW transfer and *in vivo* treatment of CRH in black porgy suggesting that the *crh* mRNA in the gill is independently functioned and regulated during environmental stress. Increased transcripts of *crh* mRNA in the gill by *in vitro* and *in vivo crh* treatment provide evidence that the *crh* may be regulated by itself in an autocrine manner. In addition, the increased expression of *a-nka* mRNA with differential localization of a-Nka cells in the filament and lamellae at 8 h and day 1 in FW fish suggests that a-Nka cells play a key role in ionic homeostasis during FW acclimation. The changes in the localization of the a-Nka cells in the black porgy gill adaptively respond to changing levels of environmental ions. Thus, the presence of *crh* and *crhr* in fish gill is essential to respond to the ambient salinity and/or stress to maintain the ion/base regulation as well as homeostasis.

## Data Availability Statement

The raw data supporting the conclusions of this article will be made available by the authors, without undue reservation.

## Ethics Statement

The animal study was reviewed and approved by the National Taiwan Ocean University (#99026).

## Author Contributions

C-FC conceived and designed the project, and supervised, wrote, reviewed, and edited the manuscript. AA and C-FC wrote the manuscript. AA, T-PW, J-CC, D-SL, and GN collected, measured, and analyzed the sample. AA and GN wrote the first draft of the manuscript. AA, GN, and C-FC worked for the revision. All authors contributed to the article and approved the submitted version.

## Conflict of Interest

The authors declare that the research was conducted in the absence of any commercial or financial relationships that could be construed as a potential conflict of interest.

## Publisher’s Note

All claims expressed in this article are solely those of the authors and do not necessarily represent those of their affiliated organizations, or those of the publisher, the editors and the reviewers. Any product that may be evaluated in this article, or claim that may be made by its manufacturer, is not guaranteed or endorsed by the publisher.
